# Compound, Comminuted, Complicated and Impacted Fracture of the Right Femur, at Junction of Upper with Middle Third, Etc.

**Published:** 1865-01

**Authors:** A. J. Baxter

**Affiliations:** Chicago


### EDITORIAL AND MISCELLANEOUS,

### COMPOUND, COMMINUTED, COMPLICATED, AND IMPACTED FRACTURE OF THE RIGHT FEMUR,

*At Junction of Upper with Middle Third—Secondary Hemorrhage—Ligation of Femoral Artery one and a half inches below Poupart's Ligament—Recovery.*

By A. J. BAXTER, M. D., of Chicago.

A recent writer says, "No humane, enlightened, and conscientious surgeon will ever resort to amputation without being satisfied of its entire and perfect necessity." Such a statement at first sight might seem superfluous, but when it is remembered that limbs have been sacrificed for selfish motives; for the glitter of operation, or to save time and trouble in subsequent treatment, we are not so much astonished, but rather blush for shame.

But the case is quite different when a member has been crushed by the passage of a railway car, or heavily-loaded wagon, or fall of an embankment. Here there is no debatable ground; but one alternative—amputation. Even under these circumstances it is a sad thing to lose a limb, but it is infinitely more deplorable to die; yet I have seen one instance where the unfortunate preferred to die with four limbs than to live with three.

The following case is interesting in several particulars:—1st, In embracing nearly all the varieties or general division of fractures. 2d, In being attended by rupture of the femoral vein. 3d, With secondary hemorrhage.

John L——, shipping clerk, æt. 40; active and stout. Nov. 1st, he was engaged in loading a barrel of flour into an express wagon, when from some cause, the horse took fright and ran. Acting on the impulse of the moment he ran and caught the horse by the bridle, but was unable to maintain his hold, fell under the horse, was trampled on, kicked, and passed over by the wheels of the wagon. Finding himself unable to rise he was picked up by those who witnessed the accident, and carried into the store in which he was employed, and that skillful surgeon, Dr. Daniel Brainard, summoned, but being otherwise engaged, very kindly referred the case to me, for which I now tender my thanks.

On my arrival at his place of business I found him propped up against the wall, and remarkably composed for one who had just gone through what the bystanders said was "enough to kill forty men." The proper mode of procedure was so apparent that I informed him as soon as possible without appearing hasty, that it would be necessary to amputate the limb, which seemed to surprise him so much that he requested me to defer the operation until he could see his family physician, to which I readily consented, and gave direction to have him taken home in a hack immediately. Having obtained several assistants I repaired to his residence and found the family physician had not arrived.

On examination he was found to be very much prostrated, and the limb enormously distended with diffused blood. As his condition was rapidly becoming more unfavorable, immediate amputation was urged by all present, yet he seemed to think it not necessary. Finally the family physician came and assured him amputation was necessary; he was satisfied.

After being brought under the influence of chloroform, and the femoral artery compressed by the thumb of an assistant, the limb was amputated about 2 inches below the trochanter minor, by the circular method.

Although the operation was performed with all the celerity I possessed, compatible with safety, it was thought he would expire before its completion. On dividing the integument and muscles a perfect deluge of blood gushed out, which had escaped from the femoral vein. At this moment rapid sinking supervened. Why the escape of this confined blood should produce such a rapid and alarming prostration (if it were the cause,) is somewhat of a mystery to me. Physiologically the blood had ceased to be subservient to the wants of the system.

The operation completed, stimulants and hot broths were given, and artificial warmth applied, under which he gradually rallied, and by 9, P. M., (eight hours after the operation,) he was as comfortable as could be reasonably expected.

From this time he rapidly improved, and everything wore a cheerful aspect until the 14th day, when all of a sudden and without any assignable cause, secondary hemorrhage supervened. On my arrival the hemorrhage had ceased; he was very much agitated, pale, and bathed in a cold, clammy sweat, and constantly asking for water. The stump was distended with coagulated blood, which extended up the inside nearly as high as the pubis. Judging from appearances he must have lost between eight and twelve ounces. What should be done? So far as the present was concerned everything was safe, but how about the future? Mr. Guthrie says, "There is no precept more important than that which directs that no operation should be done on a wounded artery, unless it bleed, inasmuch as hemorrhage once arrested, may not be renewed, in which case any operation must be unnecessary." This is very true, so far as the hemorrhage is concerned, but is this all there was to fear? I think not. The tissues contained a large amount of coagulated blood, which communicated with the air; decomposition must take place; gasses be evolved and absorbed, producing pyæmiæ, or more properly speaking ichoræmiæ, and a typhoid condition more to be feared than the hemorrhage; and as there was no certainty that hemorrhage would not recur, I decided to open the flaps, turn out the coagulated blood, and search for the faulty vessel; but in spite of all that could be done consistently, it would not bleed.

Being left without any guide to the wounded vessel, the flaps were brought together, compresses wet with cold water, applied, enjoined strict rest, with elevation of the stump, gave half a grain of morphiæ, and departed, with a *faint* hope the hemorrhage would not return. But my hope was "like a dream, to wake up in disappointment." My office had scarcely been reached before a messenger entered and gasped out, "he is bleeding again."

On reaching the house found the stump to contain a large coagulum, but not so large as the former, and the blood issuing from the inner angle of the flap, this was controlled by making pressure on the femoral artery, which afforded a clue. The femoral compressed by an assistant, the coagulum was turned out, and to facilitate matters the flap split up on the inside. The pressure being discontinued, the blood was seen to pour out or well up in a continuous stream as water rising from a spring. But the tissues were in such a disorganized condition that it was impossible to secure the bleeding vessel, and as compression of the femoral artery controlled the hemorrhage, it was concluded to ligate that vessel, which was accordingly done 1½ inches below Poupart's ligament.

From this time the patient progressed favorably, and is now going about on crutches.

On dissecting the amputated member the femur was found to be broken into six pieces. The middle and largest piece was fissured or split, but held together by the periosteum. The fifth piece from above, downward, was firmly impacted into the upper end of the lower fragment. The femoral vein had a small lacerated opening in its posterior and external aspect. All in all, presenting a variety of complications rarely witnessed in a single case.

To Doctors Hunt, Slayter, and Macdonald I am indebted for valuable assistance rendered during the operation.

January 7th, 1865.

—Elmer's Hand-Book of Practice for 1865 is for sale by our principal dealers in Medical Books and Periodicals.

